# Mid-term results of two-stage revision of total knee arthroplasty using a mobile (dynamic) cement spacer in the treatment of periprosthetic infections

**DOI:** 10.3205/iprs0000122

**Published:** 2018-05-18

**Authors:** Mohamed Ghanem, Christina Pempe, Dirk Zajonz, Andreas Roth, Christoph-Eckhard Heyde, Christoph Josten

**Affiliations:** 1 Department of Orthopaedic Surgery, Traumatology and Plastic Surgery, University Hospital Leipzig, Leipzig, Germany

**Keywords:** total knee replacement, revision surgery, infection, dynamic cement spacer

## Abstract

**Introduction:** Infection of the knee joint after primary total knee arthroplasty is a serious complication. In this work, we would like to evaluate the mid-term results after two-stage revision of total knee replacement in periprosthetic infection using dynamic spacer, in particular with regard to the function of the knee joint after reimplantation.

**Patients and methods:** In this retrospective study, we included patients who were treated in our clinic between 2005 and 2013 due to infection of the knee after total knee arthroplasty. All patients included have had a mobile antibiotic-coated cement spacer implanted after surgical debridement and removal of the components of total knee replacement. Subsequently, reimplantation of total knee replacement was performed when no clinical or paraclinical signs of infection were found. We analyzed all included cases for potential reinfection, examined the range of motion of the knee joint and evaluated the Merle d’Aubigné-Postel score. Statistical evaluation was performed with SPSS 24.0.

**Results:** This study group contains 16 patients (9 women and 7 men) with an average age of 72.0 ± 8.3 years. All patients were followed up for at least 6 months with an average follow-up of 22.5 ± 16.6 months. In all patients a pathogen was isolated intraoperatively during the first-stage surgery (explantation of the knee). *Staphylococci* were detected in 94% of the cases, *streptococci* in only one patient. Reimplantation was carried out after 6.2 ± 5.2 months. The average knee flexion in the group of patients without relapse of infection was 103.3° ± 17.1°. Only 3 patients showed extension deficit of max. 20°. The Merle d’Aubigné-Postel Score was 14.4 ± 1.9.

**Conclusion:** Two-stage surgery of total knee replacement with the use of a mobile spacer has its high value in the treatment of periprosthetic infections. The mobile spacers contribute to an advantageous range of motion of the knee joint after reimplantation of a total knee endoprosthesis. However, further studies are required that compare the results after using mobile or static spacer, but with the inclusion of homogeneous patient collective.

## Zusammenfassung

**Einleitung:** Infektionen des Kniegelenkes nach primärer Implantation einer Knietotalendoprothese ist eine schwerwiegende Komplikation. In dieser Arbeit möchten wir die mittelfristigen Ergebnisse nach zweizeitigem Wechsel einer Knietotalendoprothese bei periprothetischer Infektion unter Nutzung von dynamischen Spacer auswerten, insbesondere im Hinblick auf die Funktion des Kniegelenkes nach der Reimplantation.

**Patienten und Methoden:** In dieser retrospektiven Arbeit wurden alle Patienten analysiert, die zwischen 2005 und 2013 unter der Diagnose Infektion einer Knietotalendoprothese in unserer Klinik behandelt wurden. Bei allen Patienten wurde nach einem Ausbau der Knietotalendoprothese und einem chirurgischen Debridement ein mobiler antibiotikabeschichteter Zementspacer implantiert. Nach Ausheilung der Infektion wurde die Reimplantation des Gelenkersatzes durchgeführt. Wir evaluierten die Inzidenz der Reinfektion, den Bewegungsumfang des Kniegelenkes und erhoben den Merle d’Aubigné-Postel Score. Die statistische Auswertung wurde mit SPSS 24.0 durchgeführt.

**Ergebnisse:** Wir haben die Ergebnisse bei 16 Patienten (9 Frauen und 7 Männer) mit einem durchschnittlichen Alter von 72,0 ± 8,3 Jahren ausgewertet. Alle Patienten wurden mindestens 6 Monate nachuntersucht bei einem durchschnittlichen Follow-up von 22,5 ± 16,6 Monaten. Intraoperativ konnte zudem bei allen Patienten ein Erreger isoliert werden. Die Revisionsoperation erfolgte im Durchschnitt nach 12,1 Monaten (0,3 bis 41,9 Monate). Die durchschnittliche Kniegelenksflexion in der Gruppe der Patienten ohne Infektionsrezidiv betrug 103,3° ± 17,1°. Bei lediglich 3 Patienten zeigte sich ein Streckdefizit von maximal 20°. Der Merle d’Aubigné-Postel Score betrug insgesamt 14,4 ± 1,9.

**Schlussfolgerung:** Der zweizeitige Wechsel der Knietotalendoprothese mit der Verwendung eines mobilen Spacers hat seinen hohen Stellenwert bei der Behandlung von periprothetischen Infektionen. Die mobilen Spacer tragen zu einem vorteilhaften Bewegungsumfang des Kniegelenkes nach Reimplantation einer Knietotalendoprothese bei. Allerdings sind weitere Studien erforderlich, die die Ergebnisse nach Verwendung von mobilen bzw. statischen Spacer vergleichen, allerdings unter Einbeziehung von homogenem Patientengut.

## Introduction

Infection of the knee joint after primary total knee arthroplasty is a serious complication. An incidence of 0.4 to 2.5% has been reported in literature [1], [2], [3]. With the increasing number of primary total knee replacement in industrialized countries, the number of revision surgery is increasing [4], [5].

According to literature reports, the incidence of infection after revision of total knee arthroplasty amounts up to 5% and the incidence of infection following reimplantation of total knee arthroplasty in cases of periprosthetic infection is 15–20% [6], [7]. In early-onset infection after total knee arthroplasty, joint-preserving surgery is an established concept [6], [8], [9].

In late/chronic periprosthetic infections of the knee joint, explantation and reimplantation of total knee arthroplasty, especially the two-stage procedure, is the most popular treatment strategy [6], [8]. The advantage of the two-stage revision appears to lie in a more radical treatment of the infection [10]. 

The two-stage revision surgery of a total knee arthroplasty consists of 2 steps. In the first step, the total knee endoprosthesis components are explanted, a radical debridement and radical synovectomy is performed and an antibiotic-containing cement spacer is inserted. After the infection has healed, the second step is carried out. In this step, the cement spacer is removed and a new artificial joint is implanted. The antibiotic-containing spacer used can be constructed either in a mobile (dynamic/articulating) or a static (not articulating) form. The static form ensures that the knee joint is immobilized by the resulting temporary arthrodesis. In contrast, slight movements of the knee joint are possible with the dynamic spacers. According to literature reports, there are no differences between the use of static and dynamic spacers in the treatment of chronic periprosthetic infections of the knee joints regarding healing of infection [1], [11], [2]. However, dynamic spacers are reported to be less prone to muscle atrophy, ligament shortening, and bone loss than static spacers, so static spacers are indicated only in patients with severe bone loss or concomitant soft tissue defects [2].

In this work, we would like to evaluate the mid-term results after two-stage revision of total knee replacement in periprosthetic infection using dynamic spacer, in particular with regard to the function of the knee joint after reimplantation.

## Patients and methods

**Figure 1:**
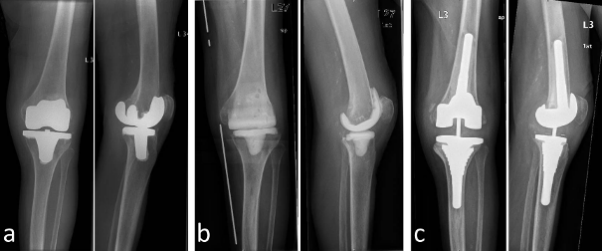
a) X-ray showing TKR with no signs of loosening, yet clinical signs of infection. b) Explantation of the components of TKR and dynamic cement spacer in situ. c) Reimplantation with a semi-constraint TKR.

In this retrospective study, we included patients who were treated in our clinic between 2005 and 2013 due to infection of the knee after total knee arthroplasty. We excluded all patients with an intramedullary cement spacer, patients who were finally treated by performing arthrodesis of the knee and all patients who could not be followed up for at least 6 months. All patients included have had a mobile antibiotic-coated cement spacer (AGC Style Company Biomet Orthopedics Inc., Warsaw, USA) implanted after surgical debridement and removal of the components of total knee replacement. The mobile cement spacer consists of a femoral component and a tibial component (each filled with 80 g cement and containing gentamycin and clindamycin) and is adapted to the anatomical condition. Broad-spectrum antibiotics were given initially and in some cases they were replaced by other antibiotics according to resistance. Systemic antibiotic treatment took place over a period of 4–6 weeks. Subsequently, reimplantation of total knee replacement was performed when no clinical or paraclinical signs of infection were found. Prior to reimplantation, a diagnostic puncture of the knee joint was performed in every case to rule out infection. Depending on the bony defect situation and the stability of the collateral ligaments, resurfacing, partially or fully constrained knee replacement was carried out (Figure 1 (Fig. 1)). We analyzed all included cases for potential reinfection, examined the range of motion of the knee joint and evaluated the Merle d’Aubigné-Postel score. Statistical evaluation was performed with SPSS 24.0.

## Results

This study group contains 16 patients (9 women and 7 men) with an average age of 72.0 ± 8.3 years. All patients were followed up for at least 6 months with an average follow-up of 22.5 ± 16.6 months. In all patients a pathogen was isolated intraoperatively during the first-stage surgery (explantation of the knee). *Staphylococci* were detected in 94% of the cases, *streptococci* in only one patient (Table 1 (Tab. 1)). Reimplantation was carried out after 6.2 ± 5.2 months. 9 patients did not have any further infection of their knee joint. In 4 patients (25%) reinfection was diagnosed and treated surgically (Table 2 (Tab. 2)). Revision surgery took place on average after 12.1 months (0.3 to 41.9 months). In one patient, the knee endoprosthesis was removed and new components were implanted in a single-stage surgery after prior arthroscopic debridement. In another patient a two-stage surgery was carried out. The two remaining patients had a poor general condition and refused surgical intervention. Therefore, we performed a stable fistula to prevent concealment of infection and systemic risks. The pathogen spectrum changed in comparison to the first revision surgery (Table 3 (Tab. 3)). The average knee flexion in the group of patients without relapse of infection was 103.3° ± 17.1°. Only 3 patients showed extension deficit of max. 20° (see Table 4 (Tab. 4)). The Merle d’Aubigné-Postel score was 14.4 ± 1.9.

## Discussion

Periprosthetic infections of the knee joint are still a major challenge in arthroplastic surgery. Several therapeutic concepts are presented in literature, but there is no consistent approach [12], [10].

In this work, we evaluated our results, particularly focusing on the functional results after the reimplantation of a total knee replacement using a mobile (dynamic) cement spacer in the treatment of periprosthetic infections. Our results are discussed in light of recent reports on the management of periprosthetic infection after total knee replacement, especially those comparing the outcome after using mobile spacers to the outcome after using static ones.

Ding et al. [13] compared the effectiveness of static versus dynamic spacers in two-stage surgery in the treatment of periprosthetic knee joint infections in a meta-analysis. They concluded that there are no relevant differences between dynamic and static spacers with regard to eradication of infection, soft tissue contractures and knee pain scores. Those patients with dynamic spacers had a better postoperative range of motion (ROM) of the knee joint. In this study, 236 dynamic spacers and 256 static spacers were included. The average postoperative follow-up was 12 months. Both the function of the knee joint and pain were evaluated according to the Knee Society Score (KSS) [14].

Citak et al. [2] compared the results of the use of dynamic spacers versus static spacers in the treatment of infection after total knee arthroplasty. The data were generated with regard to the eradication of infection and complication rate as well as the functional outcome, taking the maximum ROM of the knee joint at the time of the last follow-up examination into account. ROM was measured by the neutral-zero method. Only the studies with a follow-up period of 3 to 5 years were considered and analyzed. The collected data show no differences between static and dynamic spacers in the treatment of late-onset/chronic periprosthetic infection of the knee joint regarding eradication of infection. However, clear differences in knee joint function are seen after reimplantation inasmuch as the use of static spacers is associated with an increased risk of muscle atrophy, soft tissue contractures and bone loss. Therefore, the use of dynamic spacers is favored, except for significant bone loss and/or soft tissue defects.

Contrary to the two previously mentioned meta-analyses, Skwara et al. [15] reported no differences with regard to the final result after using static or mobile spacers. In their study, 37 cases were evaluated. All patients underwent two-stage surgery due to periprosthetic infection after total knee replacement. With regard to the ROM of the knee joints after reimplantation of total knee arthroplasty components, no difference was seen when comparing the use of static vs. dynamic spacers.

Lu et al. [8] also concluded that there is no difference in the incidence of reinfection or postoperative ROM of the knee joint with regard to the type of spacer (mobile vs. static).

However, Vasso et al. [16] reported long-term results after the use of mobile spacer in the case of two-stage replacement of a total knee endoprosthesis following infection. In their work, they evaluated the results of 46 cases with an average of 12 years (6 to 16 years). The patients were evaluated both preoperatively and postoperatively according to the International Knee Score (ICS) [14] and the ROM of the knee joint. They came to the conclusion that the use of mobile spacers leads to an advantageous postoperative result, in particular with regard to the ROM of the knee joint. However, this study is non-randomized. The results were not compared with a control group of patients with fixed spacers.

Nodzo et al. [17] analyzed in their retrospective study the results in 140 patients who had a two-stage surgery due to infection after total knee arthroplasty.

In this series, only mobile spacers were used. These mobile spacers were either prefabricated, hand-made during surgery or the femoral component autoclaved. The follow-up ranged from 43.7 to 74.9 months. Two criteria were included to define treatment success at follow-up:

- Missing clinical signs and symptoms, which necessitate new surgical intervention
- Missing clinical signs and symptoms of re-infection

The authors concluded that statistically there is no relevant difference between the different types of mobile spacers with regard to the success of treatment. However, it should be noted that the cost of the handcrafted mobile spacer is clearly lower than that of the prefabricated or autoclaved ones.

Nevertheless, subluxation is the one disadvantage of the use of mobile spacers which is frequently reported. Lanting et al. [18] reported on 58 cases in which mobile spacers have been used. The average follow-up was 44.9 months ± 29.8 months. They found that the sagittal subluxation of the spacer had a negative influence on the postoperative WOMAC scores and the Knee society scores.

Compared to the literature reports [19], [2], [13], [20], [7], [16], [9], the outcome in our series with regard to postoperative function of the knee joint is satisfactory. It should be noted that literature reports on the used cement spacers in treatment of periprosthetic infection of the knee are very heterogeneous.

The types of germs, as well as the number, age and health profile of the patients vary considerably. In addition, the follow-up duration in the different studies is very diverse.

The limitation of our study is the relatively small number of patients examined, the different germ colonization, the lack of a control group with static spacers and the fact that this is a non-randomized retrospective study.

Despite this limitation, we believe in the value of this work as we have focused on postoperative ROM and postoperative pain using of the Merle d’Aubigné-Postel score.

Different scores are used in the literature to collect postoperative results [21], [14], [22]. Therefore, a comparison of the results is difficult.

## Conclusions

Two-stage surgery of total knee replacement with the use of a mobile spacer has its high value in the treatment of periprosthetic infections. The mobile spacers contribute to an advantageous range of motion of the knee joint after reimplantation of a total knee endoprosthesis. In case of significant bone loss, static spacers are favored because they ensure stability. Generally, further studies are required that compare the results after using mobile or static spacer, but with the inclusion of homogeneous patient collective.

## Notes

### Competing interests

The authors declare that they have no competing interests.

### Authors’ statement

The authors Mohamed Ghanem and Christina Pempe have equally contributed to this work.

## Figures and Tables

**Table 1:**
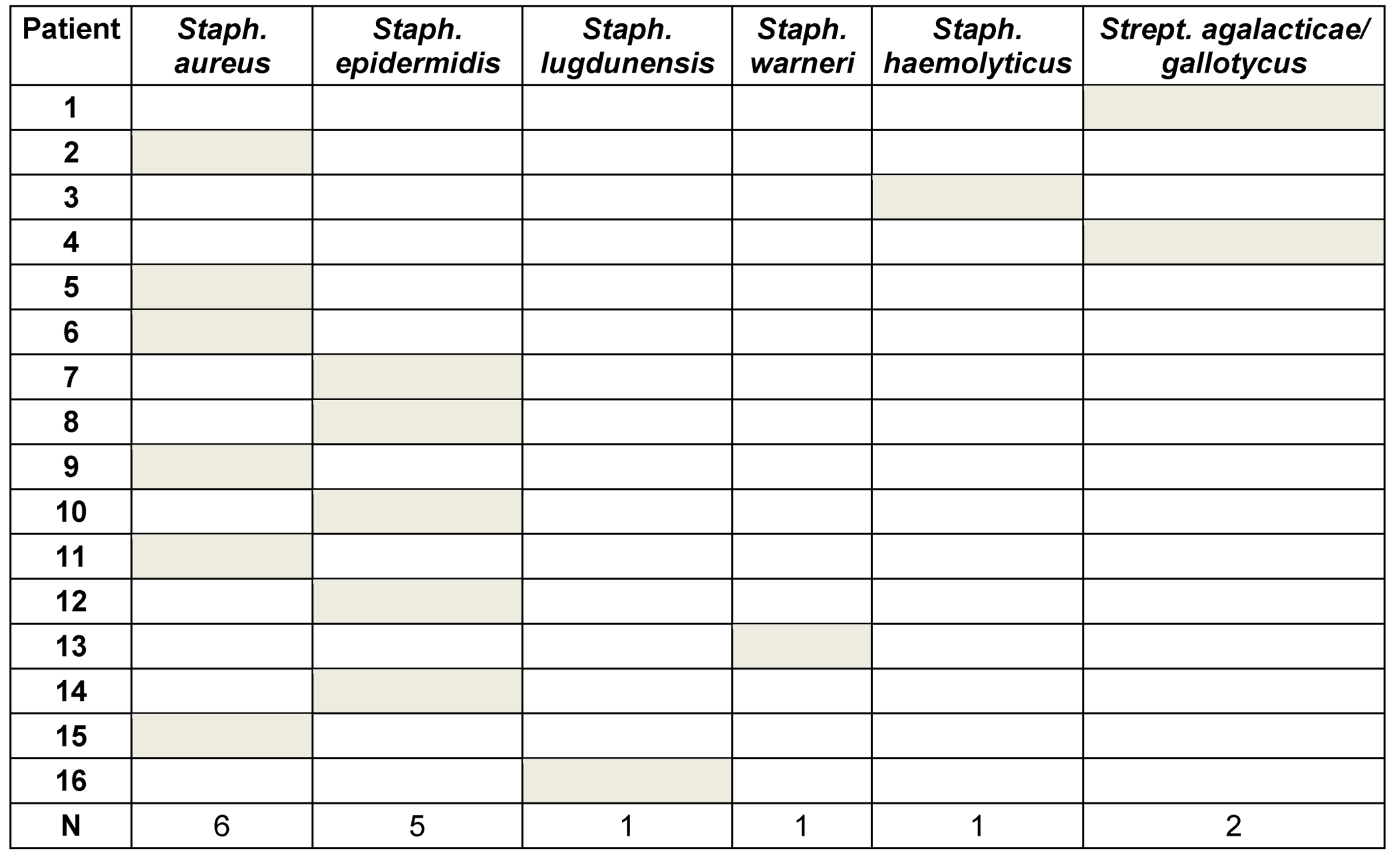
List of pathogens detected during first-stage surgery (explantation of total knee components).

**Table 2:**
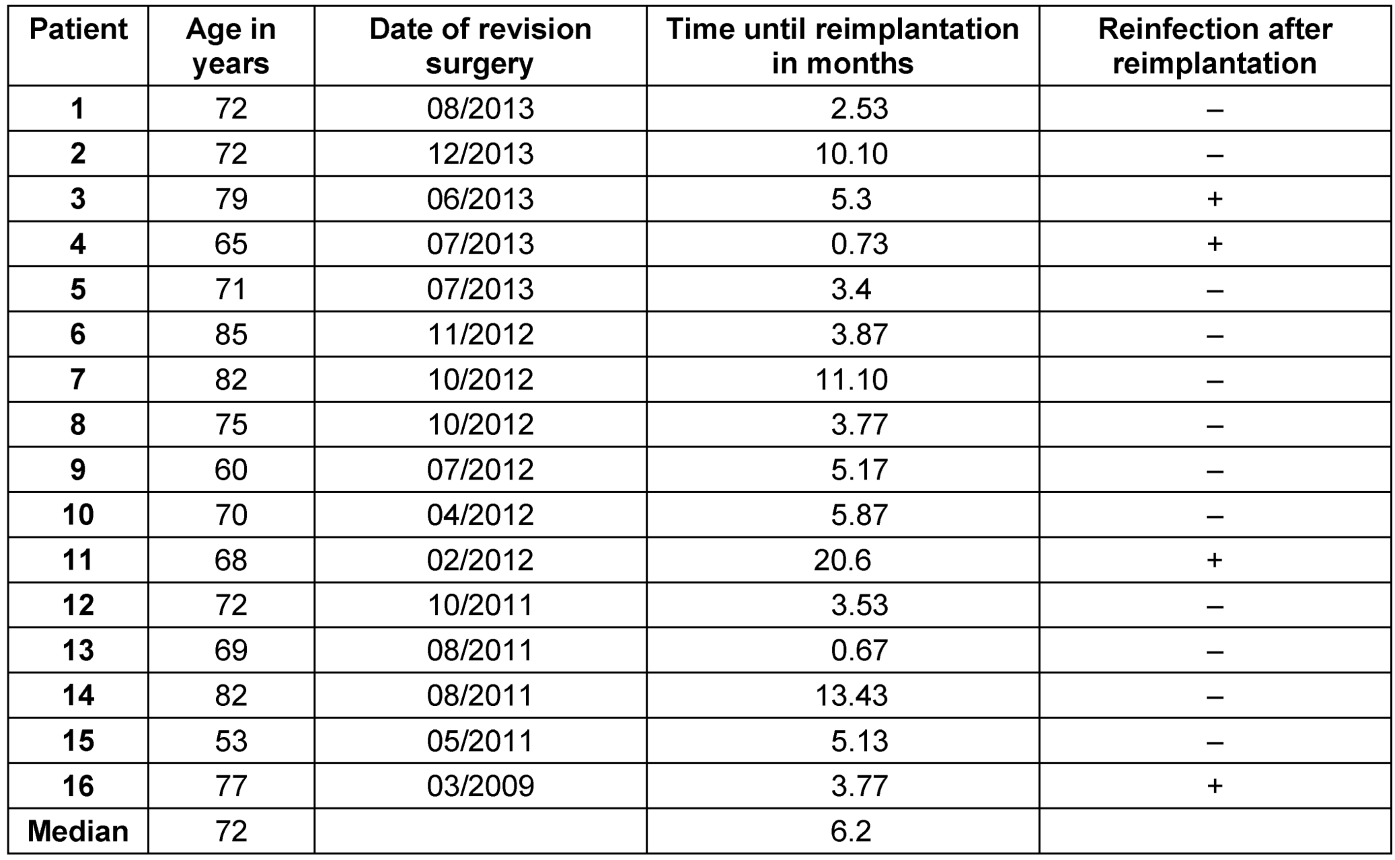
Patients’ profile of and number of reinfection occurred after two-stage surgery.

**Table 3:**
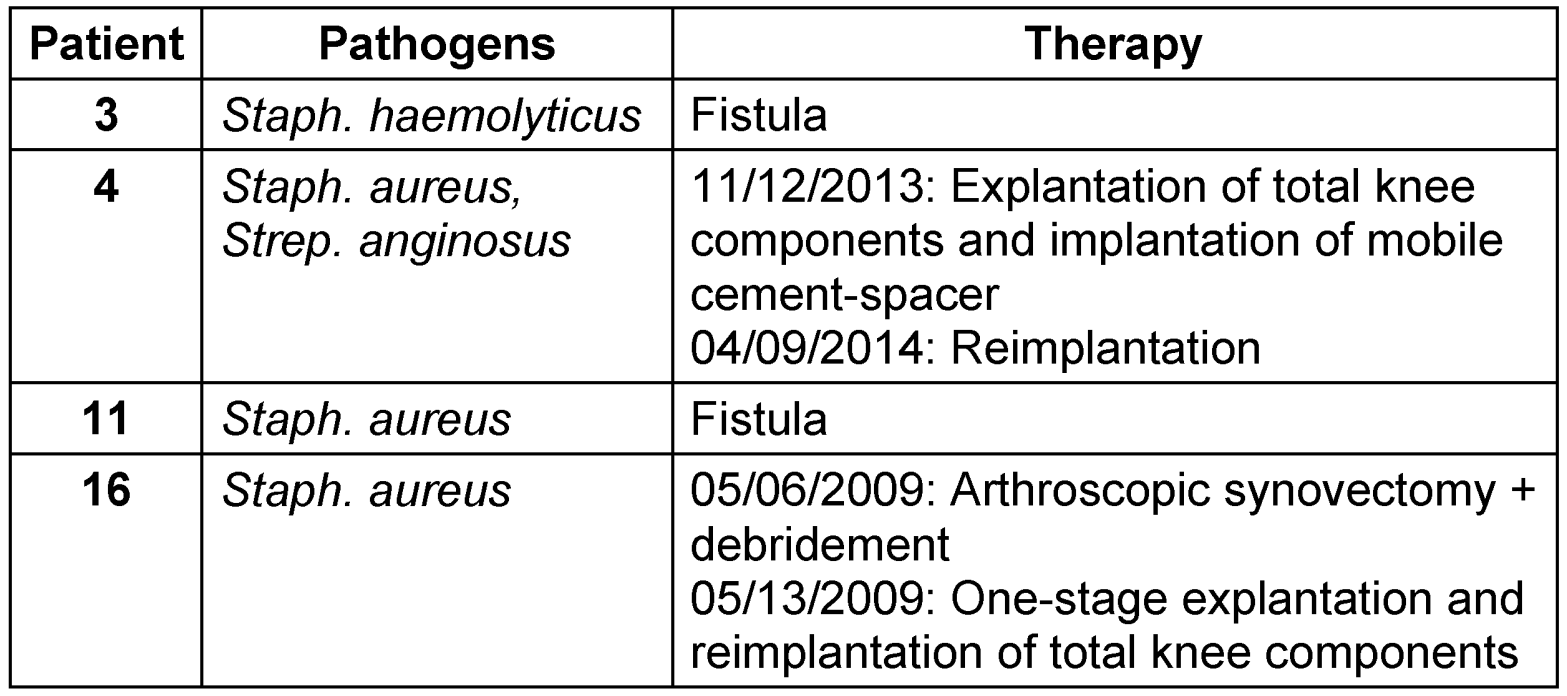
List of pathogens detected following reinfection after reimplantation and treatment of each case.

**Table 4:**
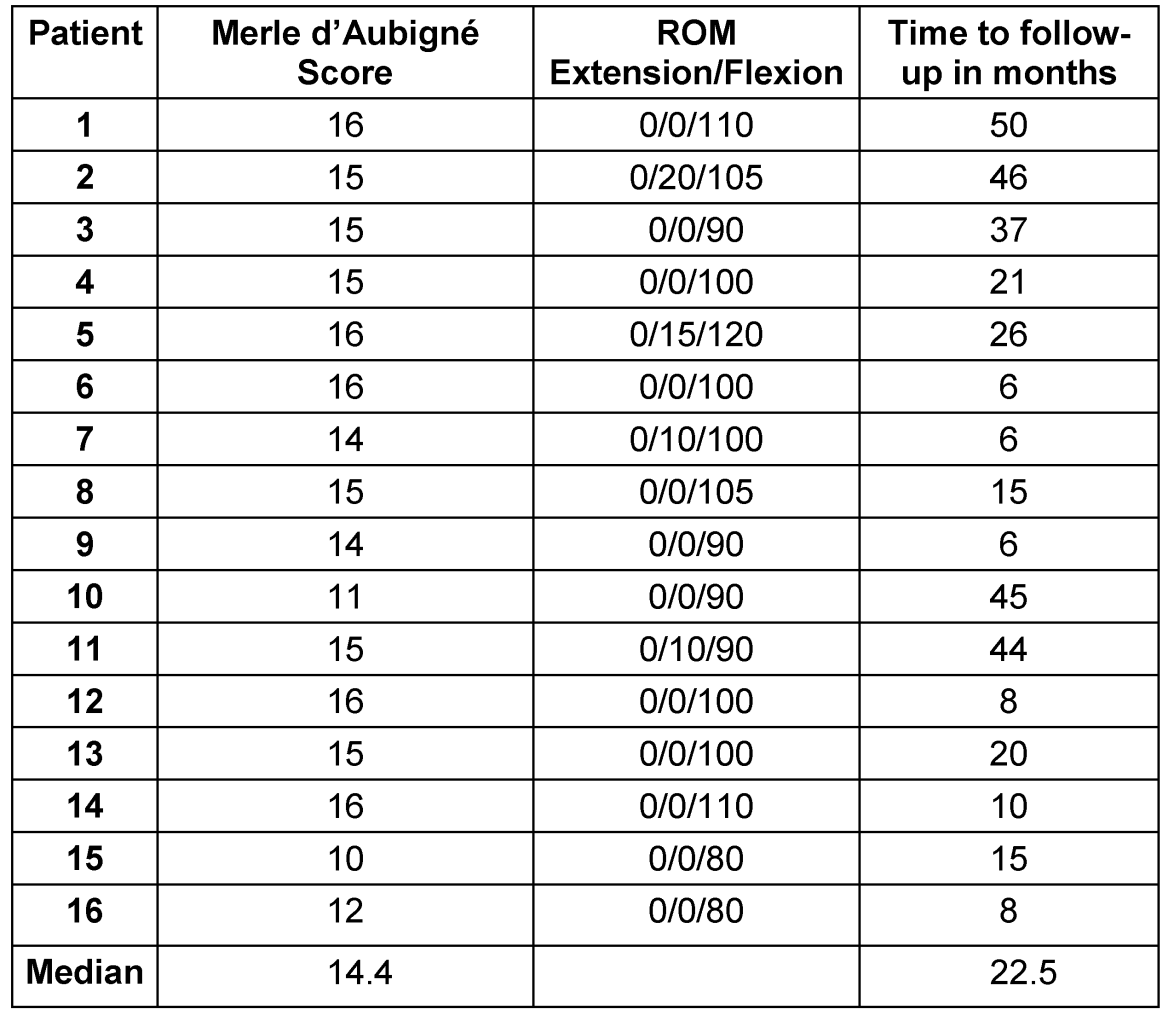
Outcome after reimplantation of total knee arthroplasty components.
